# Programmed Cell Death of Endothelial Cells in Myocardial Infarction and Its Potential Therapeutic Strategy

**DOI:** 10.1155/2022/6558060

**Published:** 2022-05-11

**Authors:** Mingyue Wu, Zixia Huang, Lijin Zeng, Chunfei Wang, Deming Wang

**Affiliations:** ^1^Department of Anesthesiology, The Second Affiliated Hospital, Hengyang Medical College, University of South China, Hengyang, Hunan 421001, China; ^2^Department of Emergency, The First Affiliated Hospital, Sun Yat-sen University, Guangzhou 510080, China; ^3^Endoscopy Center, The Seventh Affiliated Hospital of Sun Yat-sen University, No. 628, Zhenyuan Road, Guangming District, Shenzhen 518101, China

## Abstract

Cardiovascular disease, especially coronary artery disease and stroke, kills around one-third of the world's population, and myocardial infarction, a primary symptom of coronary heart disease, is a major worldwide health problem. Cardiovascular disease research has historically focused on promoting angiogenesis following myocardial damage. Myocardial vascular repair is crucial for improving myocardial infarction prognosis. Endothelial cells, the largest population of nonmyocytes within myocardial tissue, play an important role in angiogenesis. In recent years, different types of programmed cell death such as apoptosis, necroptosis, pyroptosis, ferroptosis, and autophagy have been described and found to be linked with cardiovascular diseases such as myocardial infarction, heart failure, and myocarditis. This will have important implications for reforming the treatment strategy of cardiovascular diseases. Different types of cell death of endothelial cells in myocardial infarction have been proposed, the roles and mechanisms of endothelial cell death in myocardial infarction are summarized in this review, and endothelial cell death inhibition as a therapeutic technique for treating myocardial infarction might be advantageous to human health.

## 1. Introduction

Myocardial infarction (MI) is defined as the death of myocardial cells due to insufficient myocardial perfusion, which can be caused by coronary occlusion or reduced blood flow, or by an acute supply-demand mismatch without coronary occlusion [1]. MI and subsequent heart failure are the leading causes of death in patients with coronary heart disease [2]. Perfusion of cardiac microvessels that is sustained and efficient is a significant element in avoiding cell death and extending the lifespan of injured myocardium following MI [3]. As the most nonmuscle cells in the heart, endothelial cells (ECs) play a crucial role in ischemic heart disease. The results of genetic lineage tracing revealed that newly formed capillaries after MI were generated by pre-existing ECs in the infarct marginal zone [4], and this process is facilitated by the clonal proliferation of resident ECs [3], and other cell sources have no bearing on angiogenesis.

Programmed cell death (PCD) was first used to describe cell death during insect development, and a similar process was subsequently found in humans and referred to as apoptosis. Programmed cell death is the process of removing unwanted, infected, or damaged cells and plays an important role in maintaining homeostasis of the body and host defense against pathogens. Unlike nonprogrammed cell lysis, programmed cell death is controlled by distinct signaling pathways, and these signaling pathways are cross-linked. Apoptosis, necroptosis, pyroptosis, ferroptosis, autophagy, and other kinds of PCD have all been found thus far. PCD is significant in the etiology of atherosclerosis [5], cardiovascular disease [6], and neurological disease [7]. The types of PCD of ECs in MI have been documented, but no comprehensive review has been published to date. The mechanism of distinct forms of ECs death is discussed in this review, as well as its importance in the potential therapy of MI.

## 2. Apoptosis

### 2.1. Overview of Apoptosis

**Figure 1:**
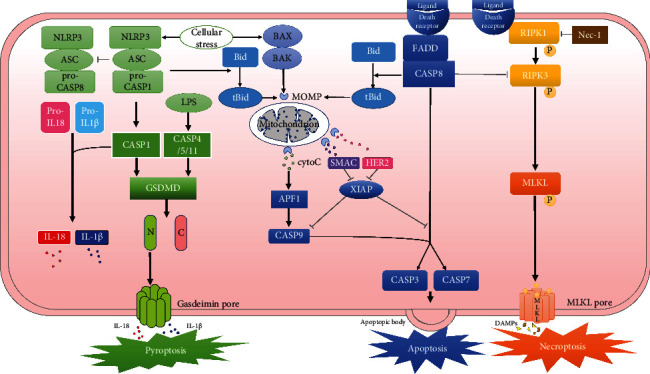
Molecular mechanisms of pyroptosis, apoptosis, and necroptosis and their network interactions. The intrinsic pathway of apoptosis (blue) is induced by the triggering of MOMP by BAX and BAK leading to cytochrome C activation and further activation of caspase-9 on APF1. Death receptor-mediated apoptosis requires the formation of a pro-apoptotic caspase-8 dimer, and both caspase-8 and caspase-9 promote the downstream executioner caspase-3 and caspase-7 activation, which induces apoptosis. Activation of death receptors similarly triggers necroptosis (orange). Activated RIPK3 phosphorylates and activates the executor of necroptosis, MLKL, which forms pores in the cell membrane, leading to cell lysis and the release of DAMPs. Cellular stress leads to inflammasome formation and activation of caspase-1 and cleavage of GSDMD, and cytokines such as IL-18 and IL-1*β* are released through the membrane pores formed by GSDMD-N and trigger pyroptosis (green).

Apoptosis, which is divided into two stages: the formation of apoptotic vesicles, and the phagocytosis and degradation of apoptotic vesicles by other cells, is a biological phenomenon first proposed by Kerr et al. [8], morphological changes such as chromatin coalescence and margination, followed by nuclear rupture, plasma membrane, or nuclear membrane wrapping broken DNA and organelles to form apoptotic vesicles, which can be quickly phagocytosed and therefore are mostly present in the cytoplasm of phagocytosed cells. Apoptosis is involved in the regulation of cell numbers in a variety of tissues, both physiologically and pathologically [8]. The intrinsic (also known as mitochondrial or Bcl-2-controlled) pathway and the extrinsic (also called death receptor) pathway both initiate apoptosis [9]. The nematode Caenorhabditis elegans expresses the human Bcl-2 gene, which decreases apoptosis [10], and ced-9 inhibits the function of ced-3 or ced-4, which has comparable effects to Bcl-2 [11]. The Bcl-2 homology 3 (BH3), Bcl-2-associated *X* protein (BAX), and Bcl-2 antagonist/killer (BAK), three functionally and physically separate subgroups of the Bcl-2 protein family, interact in the outer mitochondrial membrane and are seen as apoptosis switches. BAX and BAK produce oligomers that permeate the outer mitochondrial membrane when enough BH3-only proteins are triggered by various intracellular damages that surpass the apoptotic threshold, resulting in mitochondrial outer membrane permeabilization (MOMP) [12], which releases apoptotic factors into the cytoplasm, particularly cytochrome c [13, 14], and then, activation of caspase-9 on apoptosis activating factor 1 (APAF1) is thus promoted [15], APAF1 is homologous to ced-4 [13], and subsequent activation of caspase-3 and caspase-7 initiates the caspase cascade event, resulting in apoptosis [15, 16]. MOMP also promotes the production of second mitochondrial activator of caspases (SMAC) and HTRA serine peptidase 2 (HTR2), which both inhibit X-linked inhibitor of apoptosis (XIAP) [17] that inhibits the effector caspases [18] (Figure 1).

Another mitochondrial change is the opening of the mitochondrial permeability transition pore (MPTP), which allows nonselective passage of ≤1.5 kD molecules and causes apoptosis when the mitochondrial matrix is under high osmotic pressure [19]. Outer membrane proteins (voltage-dependent anion channel, VDAC), intermembrane proteins, at least one inner membrane protein (the adenine nucleotide translocator, ANT), and at least one matrix protein (cyclophilin D) are known to be involved in the permeability transition [20–22]. It was found that mitochondria-targeted PT inducers do trigger apoptosis in thymocytes, which is also precluded by mitochondria-specific PT inhibitors such as bongkrekic acid (ligand of ANT) [23, 24].

Apoptosis can also be triggered by the death receptor pathway (Figure 1). Tumor necrosis factor receptor superfamily, such as TNFR1, Fas, CAR1, DR4, and DR5, are related to intracellular death structural domains via their ligands [25], encouraging the development of the death-inducing signaling complex (DISC) [26], activating caspase-8, and its downstream effector caspases (caspase-3 and caspase-7) [27]. Caspase-8 also cleaves Bid, a BH3-only protein, into tBid, a pro-apoptotic form that accumulates in mitochondria and stimulates cytochrome c release, causing apoptosis via the intrinsic pathway [28].

### 2.2. Therapeutic Strategies of Inhibiting Endothelial Cells Apoptosis and Improving the Myocardial Infarction

Apoptosis was shown to be 8-9 times higher in nonmyocytes than in myocytes in myocardial biopsies from individuals with ischemic cardiomyopathy, such as myocardial infarction, and 25% of these nonmyocytes were endothelial cells [29]. Increased amounts of mitochondrial reactive oxygen species accompany cardiac ischemia-reperfusion damage, which can lead to ECs apoptosis [30]. In the mini-swine model of AMI reperfusion, Fas and Bax overexpression or Bcl-2 low expression in the infarct region and marginal tissues plays a crucial role in accelerating ECs apoptosis at Day 7 [31].

Long noncoding RNAs (lncRNAs) are important regulators of cardiac remodeling. By suppressing the miR-26b-5p/Mfn1 pathway-mediated apoptosis of cardiac microvascular endothelial cells (CMECs) following MI, the lncRNA Malat1 plays a crucial role in microcirculation repair [32]. In ECs, the long noncoding ribonucleic acid-cardiac apoptosis-related (lncRNA-CARL) can reduce the expression of Bax and PHB2, decrease caspase-3 activity, and raise the amount of the anti-apoptotic protein Bcl-2 [33]. Knocking down the lncRNA KCNQ1OT1 (KCNQ1OT1) promotes CMECs proliferation in a mouse model of AMI, while inhibiting apoptosis and lowering inflammatory factor levels [34].

Extracellular vesicles (EVs) are naturally secreted nanovesicles that play a key role in stem cell-mediated cardioprotection, and exosome production from induced pluripotent stem cells (iPSCs)-EV improves the angiogenic and anti-apoptotic properties of cardiac endothelial cells when compared to iPSCs [35]. Exosomal EV-C-MSCsN1ICD from cardiac mesenchymal stem cells reduces ECs and cardiomyocyte apoptosis mediated by oxidative stress and ischemia injury [36]. Remote ischemic conditioning (RIC)-EV inhibits ECs apoptosis by Hsp70 [37]. In vitro, miRNA-21-loaded EVs successfully transfer miR21 to recipient cells and lower the amount of the pro-apoptotic protein PDCD4; in vivo, they prevent apoptosis in ECs in the ischemia marginal zone [38]. MiR-21 prevents CMECs damage following AMI via the PTEN/VEGF pathway, according to another research [39]. MiR-24 was found to be highly expressed in cardiac ECs and was increased considerably following MI. In mice, blocking miR-24 inhibited ECs apoptosis and reduced the size of MI [40]. MiR-17-5p downregulation raises the amount of the anti-apoptotic protein Bcl-2 and lowers the level of apoptotic protein (bax/caspase 3/caspase 9) in the heart, maintaining cardiac function after an AMI by slowing apoptosis and healing vascular damage [41]. Cdip1 silencing by exosomal miRNA-21-5p-targeting cardiac telocytes prevents CMECs apoptosis and increases angiogenesis after MI [42]. MiR-124 induces ECs apoptosis via the P38/MAPK and PI3K/AKT pathways and may be a contributing factor in vascular endothelial damage in AMI [43]. By targeting granzyme B, MiR-518a-5p can reduce hypoxia/reoxygenation (H/R)-induced apoptosis and cell damage in human umbilical vein endothelial cells (HUVECs) [44].

Alpha-antitrypsin (AAT) overexpression reduces vascular endothelial cell death and increases cell proliferation under H/R conditions via decreasing the Rac1/PAK/p38 signaling pathway and antioxidative stress [45]. Fibroblast growth factor (FGF) is expressed in ECs, and basic FGF inhibits CMECs apoptosis and promotes angiogenesis via HIF-1, reducing myocardial injury [46]. CXCR7 activation has a protective impact on hypoxic ECs, reducing apoptosis, and promoting angiogenesis [47]. Under hypoxic circumstances, foxo3-mediated autophagy enhances CMECs apoptosis, which is a key pathophysiological cause of MI [48].

In addition, clinical trials have shown that the circulating apoptotic markers soluble TNF receptor 1 (sTNFR1) and sTNFR2 were found to be associated with myocardial infarct size and left ventricular insufficiency in patients with ST-segment elevation myocardial infarction (STEMI), suggesting that apoptosis may be a key determinant of the extent of I/R injury [49]. Perindopril is a third-generation angiotensin-converting enzyme inhibitor, which significantly reduces ECs apoptosis and improves abnormal ECs function in patients with MI [50]. Perindopril also reduces the pro-apoptotic effect of serum on ECs and promotes ECs renewal in patients with acute coronary syndrome [51]. The above studies suggest that therapeutic strategies targeting apoptosis have a wide range of clinical applications in myocardial infarction.

## 3. Necroptosis

### 3.1. Overview of Necroptosis

Necroptosis is a form of programmed death activated when apoptosis is inhibited, which is independent of caspase family activation pathways and can cause inflammatory responses. This process involves the activation and phosphorylation of receptor-interacting serine-threonine kinase 3 (RIPK3), which activates the pseudokinase mixed lineage kinase domain-like protein (MLKL), the final effector in necroptosis [52, 53] (Figure 1). This process promotes the release of damage-associated molecular patterns (DAMPs), which trigger inflammatory responses and the activation of pyroptosis [54, 55], as well as the efflux of pathogen-associated molecular patterns (PAMPs) [56] in the context of cellular infection, which triggers an inflammatory response. Multiple innate immune signaling pathways, including those mediated by activation of TLRs, death receptors, and RIG-I-like receptors, can cause necroptosis [57]. RIPK1's kinase activity is important in death receptor-induced necroptosis [58–60], and RIPK1 facilitates canonical necroptosis signaling when cell surface TNF binds to TNF receptor 1 (TNFR1) transduces and autophosphorylates RIPK1. Direct inhibitors of RIPK1, necrostatins, can block this pathway [57]. Intervention in the cell membrane repair process involving the ESCRT-III protein complex appears to interrupt or even prevent cell death in the early stages of MLKL activation [61]. Caspase-8 suppresses necroptosis mediated by RIPK3 and MLKL as well as acting as the starting caspase of exogenous apoptosis. The cleavage of RIPK1 by caspase-8 is a critical step in the decomposition of the death-inducing complex. TNF-induced aberrant cell death can be reduced by Asp325 of RIPK1 [62].

### 3.2. Therapeutic Strategies of Inhibiting Endothelial Cells Necroptosis and Improving the Myocardial Infarction

In CMECs, RIPK3 levels and mPTP opening rates were considerably elevated after H/R damage. Ischemia/reperfusion (I/R) injury can also activate RIPK3; however, melatonin reduces mPTP opening and prevents CMECs necroptosis after cardiac I/R injury by inhibiting the RIPK3-PGAM5-CypD signaling pathway [63]. Baicalin, a compound derived from scutellaria baicalensis, inhibits the production of the proteins RIP1, RIP3, and p-MLKL, preventing necroptosis in CMECs following H/R injury [64]. Overexpression of the sarco/endoplasmic reticulum Ca2+-ATPase (SERCA) protects I/R-treated CMECs by preventing aberrant mPTP opening and suppressing the necroptosis signaling pathway [65]. These findings imply that inhibiting necroptosis could be a useful therapeutic method for treating endothelial damage following MI.

## 4. Pyroptosis

### 4.1. Overview of Pyroptosis

The caspase-1-mediated canonical route and the caspase-4/5/11-mediated noncanonical pathway may both initiate pyroptosis [66, 67]. The caspase-1 pathway is vital in innate immunity, and the inflammasome complex signals the downstream adaptor ASC or NLRC4 to activate the caspase-1 molecule when particular stimulatory signals are detected [68], such as bacteria, viruses, and toxins [69]. The AIM2/ASC inflammasome is the most well-known, as it identifies cytoplasmic double-stranded DNA linked to AIM2; the NLRP3/ASC inflammasome is triggered by diverse membrane-damaging chemicals; the Pyrin/ASC inflammasome indirectly senses bacterial toxins inactivating host Rho GTPases; and the NAIP/NLRC4 acts as a receptor and signal amplifier for direct recognition of flagellin and type III secretion system on bacteria, respectively. The caspase-4/5/11 pathway is activated when lipopolysaccharide (LPS) is detected in the cytoplasm. The roles of caspase-4 and caspase-5 in humans are similar. Murine caspase-11 is located adjacent to caspase-1 on chromosome position, a receptor for cytoplasmic lipopolysaccharide, and a homolog of human caspase-4. Caspase-11 deletion protects mice from endotoxic shock as compared to caspase-1 deletion [70]. Caspase-4/5/11 bind directly to cytoplasmic LPS to initiate pyroptosis, and CARD on caspases mediates this binding [71]. Furthermore, Pannexin-1 channels, purinergic P2X7 pore [72], and high mobility group protein B1 (HMGB1) [73] are required for caspase-11-mediated pyroptosis and endotoxin shock [72], a mechanism that seems to be independent of TLR4 [74]. Heparin inhibits cytoplasmic transport of LPS and caspase-11 activation by reducing HMGB1-LPS interactions and glycocalyx breakdown in macrophages [75]. GSDMD is cleaved into the GSDMD-N-terminal and GSDMD-C-terminal when caspase-1 and caspase-4/5/11 are activated. The N-terminus of GSDMD is carried to the plasma membrane, where it homo-oligomerizes to form a 10- to 15-nm pore [76, 77]. This pore promotes rapid cell enlargement and membrane rupture, allowing IL-18, IL-1*β*, and HMGB1 to be released [73, 78] (Figure 1). Recent research has found that cell membrane rupture is not required for cytokine release and that IL-1*β* may be released from live cells, indicating that GSDMD might have a role in cytokine release without triggering pyroptosis [79–81]. GSDMD pores provide reasonable channels for the release of IL-1*β* (4.5 nm in diameter) and IL-18 (5.0 nm in diameter) [82]. In both mouse and human models, IL-1*β* secretion through GSDMD membrane pores has been observed [81, 83, 84], and GSDMD loss lowers IL-1*β* secretion [85].

### 4.2. Therapeutic Strategies of Inhibiting Endothelial Cells Pyroptosis and Improving the Myocardial Infarction

After myocardial infarction, ASC speck formation was observed in mouse cardiac ECs, suggesting that endothelial NLRP3 inflammasomes may play a role in MI. NLRP3 inflammasomes are involved in IL-1*β* release and pyroptosis in endothelial cells and cardiac fibroblasts [86]. Bean1 overexpression resulted in a large rise in NLRP3 and IL-1*β* in CMECs following MI damage [87], suggesting that Bean1 may serve as a therapeutic target for NLRP3-mediated vascular injury in the treatment of MI. Ventricular remodeling is a critical link in the evolution of MI into heart failure, and it is suggested that NLRP3 inflammasome also plays a significant role in the process of ventricular remodeling following MI [88]. Furthermore, by lowering vascular endothelial cell pyroptosis, chitosan hydrogel increases the efficiency of bone marrow mesenchymal stem cells in the treatment of MI [89]. The above evidence suggests that means of inhibiting ECs pyroptosis need to be discovered and applied in the treatment of MI.

## 5. Ferroptosis

### 5.1. Overview of Ferroptosis

Oxidative stress and lipid peroxidation are key determinants of cell fate, and iron-dependent lethal lipid peroxidation drives ferroptosis [90]. Glutathione peroxidase 4 (GPX4) is a selenoprotein that was discovered to cause nonapoptotic cell death due to lipid peroxidation long before the concept of ferroptosis was proposed [91]. Ferroptosis is thought to be aided by the system xc^−^-GSH-GPX4 pathway [90], with glutathione (GSH) depletion and reduced GPX4 activity affecting the metabolic process of lipid oxides, boosting the generation of reactive oxygen species (ROS) by Fe^2+^, and so inducing ferroptosis. Phospholipid hydroperoxides (PLOOHs) are major executors of ferroptosis, and GPX4 acts as a neutralizing enzyme for PLOOH, converting PLOOH to PLOH [92]. Erastin and RSL3 act as inhibitors of system xc^−^ and GPX4 to trigger ferroptosis, RSL3 directly inactivates GPX4, and erastin inhibits cystine input, reducing cysteine in glutathione GSH synthesis, indirectly reducing GPX4 production, and causing the accumulation of PLOOHs, which will lead to lipid excess. Oxidation products and an increase in oxidatively changed proteins further compromise cell membrane integrity, resulting in cell death. Acyl-CoA synthetase long-chain family member 4 (ACSL4) is a key driver of ferroptosis. Polyunsaturated fatty acids (PUFA) are the precursors of PLOOHs. ACSL4 catalyzes the linking of free PUFA with coenzyme A to generate PUFA-CoA, which is then esterified under the catalysis of LPCAT3 and forms PUFA-PL with PL. PUFA-PL is prone to peroxidation in the condition of rich iron and ROS. This accumulation of lipid peroxides in the cell membrane eventually destroys the integrity of the membrane, resulting in ferroptosis. ACSL4 depletion or inhibition drastically transforms the long-chain PUFA tails in phospholipids to short-chain or monounsaturated fatty acid (MUFA) tails [93, 94], determining ferroptosis sensitivity [95]. Ferritin heavy chain 1 (FTH1) is involved in ferritin phagocytosis and ferroptosis, and the cargo receptor NCOA4 detects and binds to FTH1 and transports iron-bound ferritin to autophagosome, a selective autophagy form, completing ferritin breakdown, and iron release [96, 97].

### 5.2. Therapeutic Strategies of Inhibiting Endothelial Cells Ferroptosis and Improving the Myocardial Infarction

Ferroptosis has been linked to the development of cardiovascular diseases [98], tumors [99], metabolic diseases [100], neurological diseases [101], and inflammatory and immunological diseases [102] in recent investigations, and ferroptosis has also been detected in cardiomyocytes from mice with MI [103]. However, ferroptosis has rarely been reported in endothelial cells of MI. Using biological information technology, Yifan et al. [104] screened the differential expression of ferroptosis-related genes in circulating endothelial cells of patients with AMI. These genes included C-X-C motif ligand 2 (CXCL2), FTH1, toll-like receptor 4 (TLR4), JUN (AP-1 transcription factor subunit), endothelial PAS domain protein 1 (EPAS1), DNA damage-inducible transcript 3 (DDIT3), Jun dimerization protein 2 (JDP2), activating transcription factor 3 (ATF3), were considerably overexpressed in the AMI group, showing that ferroptosis is involved in the control of metabolism in the circulating EC of AMI patients. This lays the groundwork for future research into the pathophysiology of ferroptosis in AMI patients.

## 6. Autophagy

### 6.1. Overview of Autophagy

Autophagy is a highly conserved adaptive mechanism that helps keep cells in a state of homeostasis. Macroautophagy, microautophagy, and chaperone-mediated autophagy [105] are three subtypes of autophagy that all entail the breakdown of cellular contents in lysosomes; however, the methods are distinct. The most common kind is macroautophagy. Macroautophagy was formerly thought to be a nonselective process, but it has now been found to selectively degrade intracellular microorganisms, damaged lysosomes, and mitochondria [106, 107]. Under cellular stress circumstances such as food restriction, oxidative stress, protein aggregation, and hypoxia, autophagy is triggered, and stress signaling pathways are mainly focused on the ULK1 complex, which is made up of four proteins, ATG101, ATG13, ULK1, and FIP200 [108]. PI3KC3 complex I, which is sorted by class III PI3K, vacuolar protein 34 (VPS34), Beclin 1, ATG14, activating molecules in Beclin 1-regulated autophagy protein 1 (AMBRA1), and a general vesicle transporter (p115), initiates phagophore nucleation [109], then comes phagophore expansion, which requires the binding of membrane-resident phosphatidylethanolamine by Ub-like Atg8 family proteins such microtubule-associated protein light chain 3 (LC3) protein and *γ*-aminobutyric acid receptor-associated protein (GABARAPs) [109]. Finally, autophagosomes are formed when a portion of the cytoplasm is phagocytosed to form double-membrane vesicles known as autophagosomes. After autophagosome maturation, the autophagosome outer membrane combines with lysosomes to produce autophagolysosomes, and the contents of the autophagosome are degraded and recycled [110].

### 6.2. Therapeutic Strategies of Inhibiting Endothelial Cells Autophagy and Improving the Myocardial Infarction

Autophagy sheds light on new therapeutic targets in the myocardial infarction. VEGF-A, ROS, GRP78/Bip, and LC3-II/LC3-I expression levels in the vascular ECs of MI mice were considerably enhanced, and VEGF-A promotes angiogenesis after AMI via the ROS-ER-autophagy axis [111]. In the injured myocardium of zebrafish, autophagy was also discovered, and metformin increased autophagic flux; caused endocardial, epicardial, and endothelial regeneration; and accelerated MI cell proliferation. When autophagic flow was stopped, however, all of these effects were delayed [112]. Angiogenic factor 1 (AGGF1) is an angiogenic factor that activates JNK in endothelial cells, causing autophagy and the formation of the Becn1-Vps34-Atg14 complex, and therapy with Aggf1 protein can greatly enhance heart function after MI [113]. Moreover, transplanted mesenchymal stem cells (MSCs) generate apoptotic bodies (Abs), which stimulate lysosomal activity and enhance the expression of transcription factor EB (TFEB), a lysosome master gene for biogenesis and autophagy, in a MI model. Following a myocardial infarction, elevated TFEB boosted the expression of autophagy-related genes in ECs, boosting angiogenesis, and cardiac function recovery [114]. To attenuate I/R-induced cardiac damage, exosomal LINC00174 derived from vascular ECs suppressed p53-mediated autophagy [115]. AMI patients exhibited considerably greater levels of sTREM-1 and significantly lower levels of Sirt6, compared to healthy controls, and Sirt6-induced autophagy was observed to restrict TREM-1-mediated pyroptosis in ECs treated with oxidatively modified low-density lipoprotein (ox-LDL) [116]. The development of drugs that enhance autophagic flow would be a potential therapeutic strategy to improve the prognosis of MI.

## 7. Cross-Talk between Apoptosis, Necroptosis, Pyroptosis, Ferroptosis, and Autophagy

It was speculated that inhibition of caspase-8 and its adapter FADD would prevent apoptosis induced by the death receptor pathway, leading to an increase in cell numbers. However, this was not the case, and the study found that necroptosis was activated after caspase 8 [117] and FADD [118] were inhibited, which is the first time that the possibility of functional intertwining of different programmed cell deaths was identified [119, 120]. Necroptosis may be a means to kill damaged cells that have evaded apoptosis. Caspase-8 not only plays an important role in apoptosis and necroptosis, but also is able to regulate pyroptosis. Procaspase-8 inactivation also leads to ASC spot production, which promotes caspase-1 activation and subsequent GSDMD lysis and pyroptosis [121]. In the later stages of apoptosis, a decrease in intracellular K^+^ concentration leads to the activation of NLRP3 inflammasome and the onset of pyroptosis [122]. Notably, cells lacking caspase 1 or GSDMD still die due to NLRC4 activation, and this death is morphologically characteristic of apoptosis [123] and involves caspase 8, which can bind to ASC or NLRC4 [124, 125]. This suggests that in the absence of GSDMD, sustained inflammasome activation, although not inducing pyroptosis, leads to apoptotic forms of cell death. Alternatively, inflammasome activation can trigger apoptosis via caspase-1 or caspase-8-mediated processing of the BH3-only protein BID into a pro-apoptotic tBID, followed by activation of BAX/BAK and induction of MOMP via the intrinsic pathway [126, 127] (Figure 1). We know that both active MLKL and GSDMD may lead to membrane damage and that MLKL-dependent K^+^ spillover leads to NLRP3 activation [128]. Ferroptosis is a ferritin-degrading autophagy-dependent process [129], and it has been reported that knockdown or knockout of ATG5 and ATG7 can prevent iron death induced by the ferroptosis-inducer erastin by reducing intracellular iron levels and lipid peroxidation [130]. In addition, autophagy can reduce intracellular GSH levels [131], so the use of autophagy inhibitors can prevent the occurrence of GSH depletion-dependent ferroptosis [132]. This indicates that autophagy plays an important role in ferroptosis.

## 8. Conclusions

As summarized herein, apoptosis, necroptosis, pyroptosis, ferroptosis, and autophagy are all seen in endothelial cells following myocardial infarction. The majority of current research focuses on a single kind of cell death, despite the fact that many types of cell death have interconnected effects [133]. Cross-talk between different modes of endothelial cell death in myocardial infarction will be an area of ongoing interest, and it is unclear whether cell death is a direct cause or a key factor in the development of disease, or a consequence of disease-induced injury, and the extent to which therapeutic strategies targeting cell death can alleviate disease remains to be explored. With a better understanding of the different cell death modalities, more research focusing on the role of endothelial cell death in the pathogenesis of myocardial infarction is needed in the future, thus providing more possibilities for the treatment of myocardial infarction.

## Data Availability

No data were used to support this study.
